# Are Lady Doctors Clever?

**Published:** 1890-10-11

**Authors:** 


### The Doctor's Pulpit.

### ARE LADY DOCTORS CLEVER?

Ladies have had their fight and established their claim to qualify and practice as doctors. That is in every way a good thing. Nothing can be more entirely stupid and ridiculous than to keep persons out of particular occupations solely on the ground of sex. The prejudice of the public and the interested motives of medical men which led them to oppose women in their efforts to study medicine were not even respectable. The non-medical man who considered it "improper" that women should work at anatomy and pathology was a stupid goose—the medical man who saw danger to his financial interests in the accession of woman to the ranks of medicine, and who strove to intensify public prejudice for his own ends, was a mean fellow. Happily the public has got rid of its prejudice, and the medical man is ashamed of his opposition; and now medical women have not only a "fair field," but a good deal of "favour."

This present writer has for twenty years—the whole of his medical life, in fact—maintained the right of women to exactly the same freedom of medical study and practice as men possess. But though he would consider it unmanly and contemptible to put a single straw of opposition in their way, he sees no reason whatever why their actual work as doctors should not be freely criticised, and put into comparison with that of men's. On the contrary, one of the best services which can be rendered to lady doctors, as to all other workers, is that of candid and impartial criticism.

One of the first things that strikes a man in taking a general survey of medical women and their work is that the department of surgery seems to be altogether run away from by lady doctors. But surgery is the very part of medicine which offers the most definite work, and which furnishes the most definite and easily judged results. To surgery mainly, and to pure medicine very little, is due the high esteem in which medical practice is held to-day as compared with a hundred years ago. The physicians of the day have, we think, advanced quite as much as the surgeons. But their work is of such a kind that neither the medical man himself nor the public can see cause and effect in it with so much definiteness and precision as in surgery. When we say that the surgeons have done most to raise medical men in the popular esteem, we merely mean that they have had the good fortune to do work which the public could easily understand; whilst the less fortunate physicians have been, like the industrious mole, or the germinating acorn, compelled to carry on their operations largely under ground. But although surgery would thus seem to have offered to women a more favourable field for displaying their digital skill and neat handiness than pure medicine, it yet remains the fact that they have, almost by universal consent, boycotted surgery.

This is not at all satisfactory. It does not please the friends of medical women; and it ought not to please medical women themselves. There are several operations constantly performed, which, by this time, should have been almost exclusively in the hands of women. Ovariotomy, for example, only needs to be mentioned as a case in point. Yet, whilst we have in this country such famous ovariotomists among men as Sir Spencer Wells, Thomas Keith, Lawson Tait, Knowsley Thornton, Granville Bantock, and others, we know of no single woman who has even attempted to achieve fame in what ought to be so peculiarly a woman's operation. This is but a sample of what is happening with regard to all the greater operations in surgery. They are left to men. Women seem as if they dare not look at them. But, supposing men had shown the same timidity—or is it disgust?—at surgery, where would the whole art of medical and surgical practice have been to-day? Women may depend it that they will be judged by their all-round capacity for medical practice. They will never be considered the equals of men unless they can practise surgery equally well. They may be allowed to obtain qualifications; and their success in reading books and committing facts to memory may be applauded, but there is nothing more certain than this, that it is in the arena of actual life-work and practice that women as well as men will be tried. They will stand or fall, not by their academic proficiency, but by their homely handiwork.

Although surgery and a particular surgical operation have been cited as instances of the deficient practical capacity of medical women, yet it does not appear to be in medicine alone that they are inferior to men. The reason why the writer has been led to consider the subject at the present time is that several ladies who have been mistakenly treated by members of their own sex in the region of pure medicine have come under his notice during the last few months. In more than one instance girls who have had nothing more the matter with them than spring coughs have been advised to give up profitable occupations on the ground of impending phthisis. Now this is a serious business. To any one accustomed to the skilled use of the stethoscope there ought to be not the slightest difficulty in differentiating between a simple cough caused by an irritable throat or bronchial tubes and the commencement in the apices of the lungs of a phthisical process. The lady doctors may think that a mistaken diagnosis in the case of a young girl is a matter of very little importance. But what will the girl herself think? And what will her parents think; or her lover, if she has one? A mistake of this kind may not only cost a girl a profitable situation, but a husband also; for no man ought to marry a woman in whom consumption has commenced. A girl may thus have all her prospects blighted and her life made miserable for an affirmed reason that does not exist.

Of course, we may be met by the argument that men fall into errors of diagnosis as well as women. That is most true. But the question is—do they fall into such errors as frequently as women? We think not. At present the facts about lady doctors seem to be these: that they have studied diligently, and passed their examinations, on the whole, well; but that in actual practice they are less thorough, less courageous, and less capable than men. Medicine, indeed, is a homely kind of business, having little flavour of poetry about it, and not much of academic refinement and nicity. Most of the illnesses that afflict human bodies are of a homely type, and require for their management plain sense, ready resource, a kind and motherly helpfulness. Medical women are apt to take a strained, exalted, and far-off view of things. The working for a degree has been the supreme effort to them; and they incline to regard the commonest incidents of every-day practice from a supreme and impractical point of view. Men, who get their degrees with less of mental strain, take things more naturally and quietly; and so their measure is juster, their confidence greater, and their resources readier and more efficient. In what we have said we have been animated by the desire of helping women to take a true view of the situation. Can they bring their actual work to the level of the all-round requirements of good medical and surgical practice? If they can they may properly continue to seek medical qualification. If they cannot they will be well advised to let medicine alone.

